# Multi-step pre-treatment of rice husk for fractionation of components including silica

**DOI:** 10.3389/fchem.2025.1538797

**Published:** 2025-01-23

**Authors:** Shinnosuke Ishida, Shinji Kudo, Shusaku Asano, Jun-ichiro Hayashi

**Affiliations:** ^1^ Interdisciplinary Graduate School of Engineering Sciences, Kyushu University, Kasuga, Japan; ^2^ Institute for Materials Chemistry and Engineering, Kyushu University, Kasuga, Japan

**Keywords:** biomass, rice husk, fractionation, pre-treatment, silica

## Abstract

**Introduction:**

Rice husk, a widely available agricultural by-product lignocellulosic biomass, is a promising and sustainable feedstock for organic and inorganic chemicals due to the rich silica content. However, its current application is largely limited, with most being incinerated as waste. This study introduces a novel multi-step pre-treatment process to fractionate these components efficiently, enabling their comprehensive valorization.

**Methods:**

The process begins with hydrothermal treatment, selectively extracting hemicellulose while preserving the structural integrity of other components. This is followed by an organosolv treatment using ethanol/water mixture, optimized to dissolve and extract organosolv lignin effectively. Subsequently, alkaline aqueous solution treatment under nitrogen atmosphere facilitates the recovery of silica as sodium silicate, a valuable industrial precursor. To ensure high-purity cellulose recovery, the final step employs alkaline hydrothermal processing under oxygen, achieving effective lignin depolymerization and removal.

**Results and discussion:**

Each step was carefully optimized, considering parameters such as temperature, solvent composition, and reaction time, to enhance selectivity and yield. Notably, this method reduces environmental negative impact by avoiding the use of acids while utilizing renewable solvents. The sequential application of these treatments resulted in separation exceeding 97% for hemicellulose, lignin, and silica, leaving high-purity cellulose with the loss of 22.8 wt%. Hemicellulose, organosolv lignin, and silica in the removed fractions were 66.5, 78.1, and 77.5 wt% at the first, second, and third treatments, respectively.

## 1 Introduction

Lignocellulosic biomass has garnered attention as a renewable resource that can be converted into a variety of chemicals. Cellulose is the most valuable component in lignocellulose and can be directly or indirectly via its hydrolysis product, glucose, converted into a variety of compounds and materials (Delidovich et al., 2014). Similarly, hemicellulose, another carbohydrate, is expected to be used as a raw material for compounds such as furans or as xylooligosaccharides, food ingredients (Santibanez et al., 2021). Lignin is the only direct and renewable feedstock for aromatic compounds, and its conversion into phenolic compounds or materials is being actively studied (Li et al., 2024). To establish an economically viable biomass chemical industry, it is crucial to efficiently separate these components (Xu et al., 2020), which are intricately intertwined chemically and physically in biomass.

Among lignocellulosic biomass, rice husk is particularly notable for its high ash content, especially silica (Zhang et al., 2022). Rice is the third most produced crop globally after sugarcane and maize, and it constitutes 20%–22% of the total rice weight, generating over 100 million tons annually worldwide (Soltani et al., 2015; Bazargan et al., 2015). Thus, rice husk, containing 15–20 wt% silica, holds potential not only as feedstock for organic chemicals derived from the three main organic components but also as a raw material for inorganic chemicals. The ash in rice husks is being explored for use in various industrial fields, including rubber, electronics, concrete, soil conditioners, and catalyst (Soltani et al., 2015; Moayedi et al., 2019). Research and development on its use as an additive in tires are highly active (Shiva et al., 2024). However, rice husk utilization currently remains limited, with most being incinerated as waste, except for small quantities used as livestock feed (Bazargan et al., 2015). Furthermore, research on application development often focuses solely on extracting silica, leaving organic compounds to be incinerated. Technologies for valorizing all four major components in rice husk, hemicellulose, lignin, ash (silica), and cellulose, through an effective fractionation would contribute to its broad utilization due to sustainability and economic advantages.

Numerous methods for selective fractionation of biomass components have been reported (Taylor et al., 2015). For instance, the traditional kraft pulping process removes lignin using alkaline aqueous solutions to obtain pulp (cellulose). In the “lignin-first strategy,” which is a concept emerging during this decade, lignin is depolymerized and hydrogenated in organic solvents to produce lignin monomers alongside pulp. Conversely, in common methods for quantitatively analyzing components composition of lignocellulose (Sluiter et al., 2011), carbohydrates are firstly removed through concentrated and dilute sulfuric acid treatments, leaving lignin as solid residue (acid-insoluble lignin). There have been some pre-treatment technologies having technology readiness level of demonstration or commercial stage such as steam explosion, dilute acid pre-treatment and water-solvent pre-treatment (Gutierrez et al., 2023).

In the present study, a multi-step pre-treatment consisting of hydrothermal treatment, organosolv (ethanol/water) treatment, and alkaline aqueous solution treatments was examined to achieve the fractionation of four components in rice husk. Although acids are often used in the component separation, they pose environmental problems, such as reactor corrosion and waste liquid disposal, and cost challenges. Hydrothermal reactions using only water are worth considering. Kumagai et al. (2004) applied the hydrothermal extraction with a two-step process at 200°C and 260°C in a percolation type reactor and obtained holocellulose with the yield of 84.3 wt%. Organosolv treatment, initially developed as an alternative to the kraft process for pulping, has seen extensive research, employing various organic solvents such as alcohols (methanol, ethanol, butanol, glycerol, ethylene glycol), ethylene carbonate, organic acids, and acetone (Sun et al., 2023). Its attractive feature is the ability to separate organosolv lignin (El Hage et al., 2009) without using alkali metal salts. The present study adopted ethanol, a solvent easily available from biomass, and used it as a mixed solvent with water to adjust the solubility parameter for organosolv lignin extraction. Various reports exist on ethanol/water extraction treatments; for instance, Di Francesco et al. (2021) employed an OrganoSoxhlet reactor with CO_2_ as an acid catalyst to treat sugarcane bagasse and wood, achieving high lignin extraction rates. Alkaline aqueous solutions are used for silica extraction and lignin removal, where the latter is as the finishing step for purifying the pulp. In industry, the treatment of feedstock with NaOH aqueous solution is used to produce sodium silicate, which is then converted to silica product with desired properties (Liu and Ott, 2020). Lignin extraction was conducted by adding oxygen to enhance removal rates. Bleaching with oxygen is used in the pulping process, which enables the removal through benzene ring opening and carboxyl groups formation by oxygen ions (O_2_^−^) (Ohmura et al., 2012; Wang et al., 2021; Wang et al., 2020).

In this work, first, the effects of reaction conditions in ethanol/water treatment on the removal of each component was investigated. Based on the findings, the treatments with alkaline aqueous solutions were also incorporated to attempt the fractionation of rice husk into four components. While combining known treatments, this study uniquely aimed to construct a rational multi-step process including silica extraction. Additionally, water and ethanol/water treatments were conducted under a pressurized CO_2_ to explore its influence on the fractionation performances.

## 2 Methods

Rice husk, sourced from Ishikawa Prefecture, Japan, was used as the raw material after crushing to under 3 mm. The component composition of the raw material and solid residues generated through pre-treatment was analyzed following a reported method (Sluiter et al., 2011). Briefly, rice husk, extracted with water and then benzene/ethanol, was treated with 72% sulfuric acid (30°C, 1 h), then dilute sulfuric acid (4%, 121°C, 3 h), followed by solid-liquid separation. Lignin in the filtrate was quantified as acid-soluble lignin (ASL) using a UV-vis spectrophotometer (PerkinElmer, LAMDA365). Acetyl content was analyzed using liquid chromatography (Shimadzu, Prominence Series) with a BIO-RAD Aminex HPX-87H column, and saccharides were analyzed with a Shodex SPO0810 column. For saccharides analysis, pH of the filtrate was adjusted to 5–6 using barium carbonate octahydrate. The solid residue was dried, weighed, and classified as acid-insoluble lignin (AIL). Ash content was analyzed by ashing through gradual heating to 575°C with intermediate steps at 105°C and 250°C, in a muffle furnace (Sluiter et al., 2008). The composition of rice husk ash was analyzed using an energy-dispersive X-ray fluorescence analyzer (Malvern Panalytical, Epsilon 1).

Tables 1, 2 show the component composition and ash composition of the feedstock rice husk, respectively. Hemicellulose in herbaceous biomass is comprised primarily of arabinoxylan (Farhat et al., 2017). Xylose and arabinose were indeed detected in the filtrate of sulfuric acid-treatment. Cellulose and hemicellulose contents were calculated based on quantified glucan and other saccharides (xylan, arabinan, and galactan), respectively. The rice husk contained 20.5 wt% ash, with 93.6 wt% of it being silica.

**TABLE 1:** Composition of rice husk.

Component		wt%-dry
Cellulose	Glucan	26.2
Hemicellulose	Xylan	11.2
	Arabinan	1.3
	Galactan	0.8
	Acetyl	4.0
Lignin	ASL	0.8
	AIL	21.6
Ash		20.5
Extractives		13.6

**TABLE 2:** Composition of ash in rice husk.

Component	wt%
SiO_2_	93.6
K_2_O	4.7
CaO	0.8
MnO	0.2
Fe_2_O_3_	0.1

For all pre-treatment experiments, an autoclave (Taiatsu Techno, TPR1-VS2-500, 500 mL) was used. 10 g of rice husk or solid residue obtained from treatment was mixed with 200 mL of solvent. Pressure inside the reactor was set to 0.8 MPa with a filling gas (CO_2_, N_2_, or O_2_) after repeated purge. The slurry was heated, while stirred at 200 rpm, to a prescribed temperature, which was maintained for 1–4 h. Time zero was defined as the moment, where the target temperature was reached. The heating rate was approximately 15°C/min. The reaction was conducted under autogenous pressure formed by the saturated vapor of water. After treatment, the autoclave was cooled with ice water, and the slurry was filtered using a PTFE membrane filter. The filter cake was vacuum-dried (50°C, overnight) to obtain solid residues. The removal or extraction yields of components from solids were determined from the result of compositional analysis. All compositions and yields were calculated on a dry mass basis.

Some experiments also involved quantifying mono-saccharides and oligo-saccharides in the extracts. The filtrate was subjected to liquid chromatography analysis directly for analyzing mono-saccharides and after hydrolysis with diluted sulfuric acid for analyzing all saccharides. The extraction yield of oligo-saccharides was calculated by subtraction.

## 3 Results and discussion

### 3.1 Ethanol/water treatment

**FIGURE 1:**
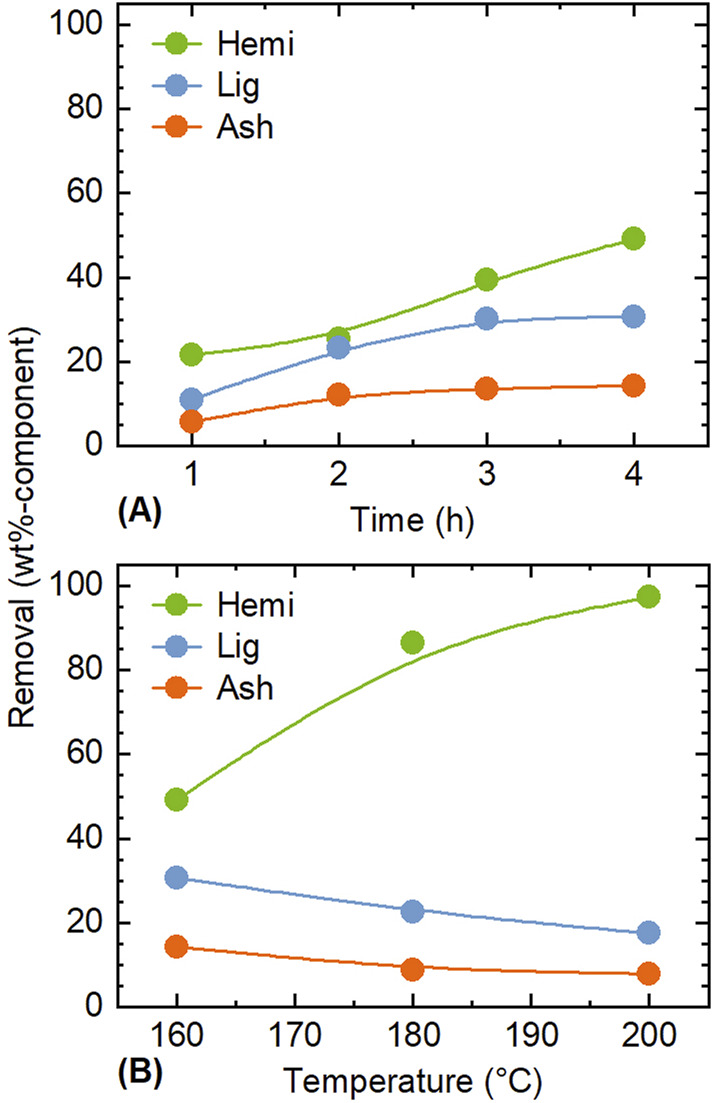
Influence of **(A)** time and **(B)** temperature on the removal of each component during the pre-treatment with 25 vol% ethanol/water. Conditions: rice husk 10 g, ethanol/water 200 mL, CO_2_ 0.8 MPa, **(A)** 160°C, and **(B)** 4 h.

The influence of conditions for treatments using ethanol/water as solvents on the removal of each component was analyzed. Figure 1 shows the effects of time and temperature on the removal of hemicellulose, lignin, and ash during the treatment with 25% ethanol/water. Because cellulose was hardly extracted under the conditions applied in this experiment as shown in the next section, its removal is not presented here. For the treatment at 160°C and short time (≤2 h), the removal of all components was well below 30 wt%, and there was no significant difference in the removal rates among them (Figure 1A). This suggested that each component contains fractions that were readily dissolved in the solvent. For ash, the removed fraction should include alkali and alkaline earth metallic species (AAEMs) in the form of salts.

Figure 1B compares treatments at different temperatures at the fixed time of 4 h. The removal of hemicellulose became marked with increase in the temperature, reaching 97.3 wt% at 200°C. This aligns with the general trend that hemicellulose is the most easily extracted component under hydrothermal conditions (Yu et al., 2007). In contrast, the removal rates of lignin and ash decreased with temperature. The decrease in lignin removal is attributed to the re-polymerization, condensation and precipitation of dissolved organosolv lignin and possibly hemicellulose (Wang et al., 2018). A similar phenomenon is thought to occur with ash, although the degree of decrease in removal is smaller (from 14.4 to 7.9 wt%), compared to lignin (from 30.7 to 17.6 wt%). Di Francesco et al. (2021) reported that treatment of woody biomass (Populus) with a 20 vol% ethanol aqueous solution at 220°C for 4 h reduced the residual lignin to 3 wt%. Thus, higher temperatures may further improve lignin removal; however, higher temperatures may also lead to partial removal of cellulose. Because the removal of cellulose indeed increased to 3.7 wt% at 200°C, this study did not examine the treatment at higher temperatures.

**FIGURE 2:**
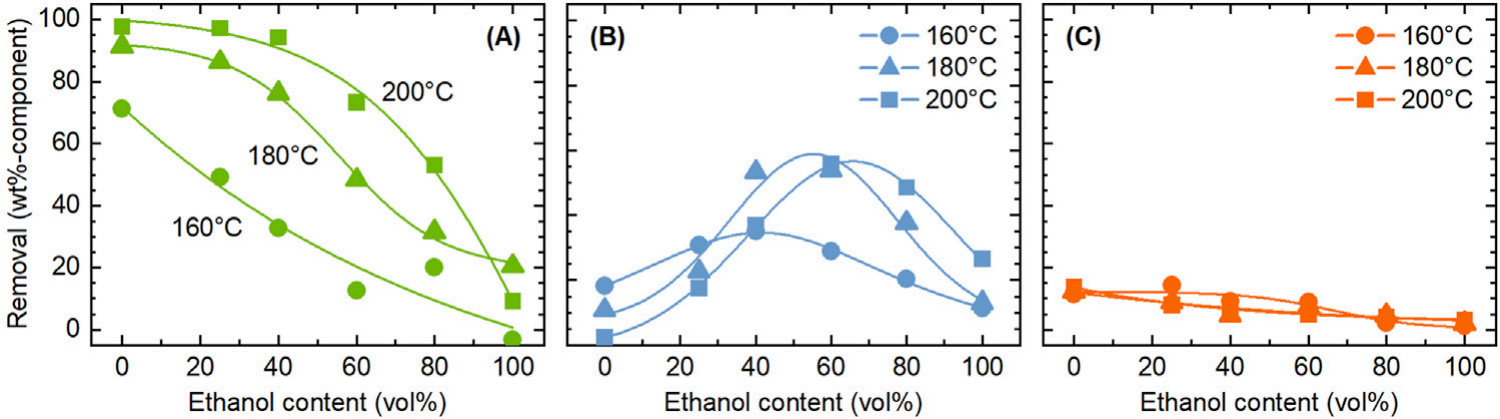
Influence of temperature and ethanol concentration on the removal of **(A)** hemicellulose, **(B)** lignin, and **(C)** ash, during the pre-treatment with ethanol/water. Conditions: rice husk 10 g, ethanol/water 200 mL, CO_2_ 0.8 MPa, and 4 h.

Figure 2 shows the influence of reaction temperature and ethanol concentration on the removal of each component. The cellulose removal in this test was equal to or below 3.7 wt%, confirming that cellulose extraction was suppressed under employed conditions. The highest removal of hemicellulose was obtained in the absence of ethanol at 200°C, reaching 97.8 wt%. At all temperatures, increasing the ethanol concentration reduced the hemicellulose removal. The removal was below 20 wt% in ethanol-only conditions. Therefore, water-only system was concluded to be appropriate for hemicellulose extraction.

Ash removal also tended to decrease with increasing ethanol concentration, although the removal level was low, 13.7 wt% even at the highest case, indicating that the amount of ash soluble in ethanol/water system is limited at temperatures up to 200°C. Since silica is insoluble in water, most of the removed ash is inferred to consist of other metal species. However, silicon exists in rice husk in various forms, such as silicic acid that is soluble to water (Vassilev et al., 2013). Vassilev et al. (2012) analyzed the water solubility of ash metal species in various biomass types, finding that 2% of silicon was leached to water in average. The soluble portion of potassium was as high as 71% in average, the second element most easily leached into water after chlorine.

Unlike other components, lignin had an optimum ethanol concentration (40–60 vol%) to be dissolved, with the highest removal of 55.9 wt%. This result can be explained by the solubility parameter. In general, polymers and macro-molecules like lignin exhibit higher solubility in solvents with solubility parameters (δ values) similar to their own. Ni and Hu (2003) experimentally demonstrated that the solubility of Alcel lignin in ethanol/water mixed solvents at room temperature reached its maximum at an ethanol concentration of 71.3%, corroborating this finding through δ value calculations for both the solvent and lignin. Renders et al. (2016) also reported that lignin solubility in ethanol/water was highest at 50% ethanol concentration during catalytic reductive fractionation of woody biomass. Considering that lignin structure and solvent temperature also affect δ values, the results obtained in this study are deemed reasonable.

**FIGURE 3:**
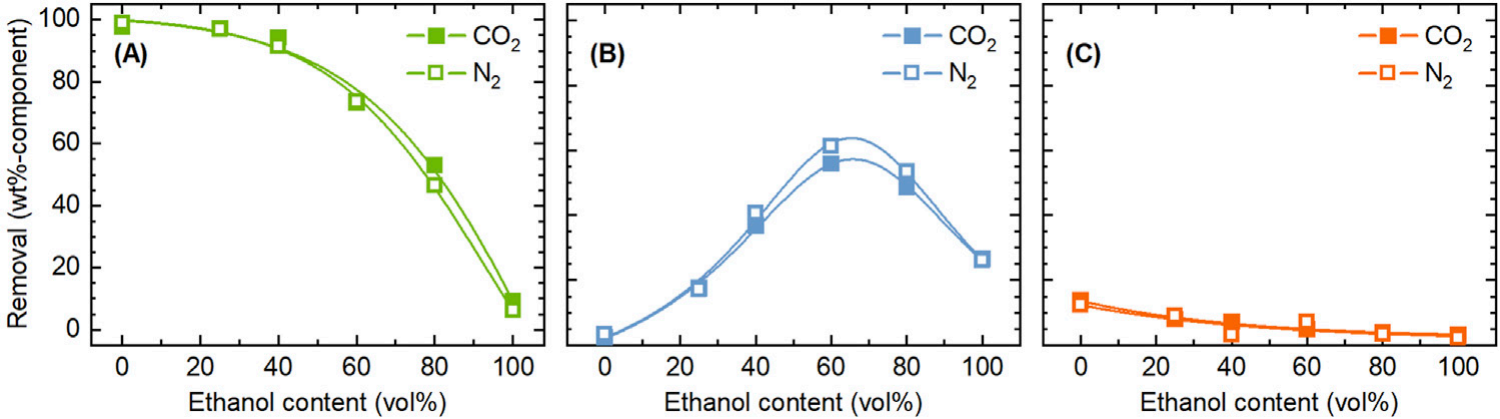
Comparison of CO_2_ and N_2_ as filling gas in terms of the influence on the removal of **(A)** hemicellulose, **(B)** lignin, and **(C)** ash, during the pre-treatment with ethanol/water. Conditions: rice husk 10 g, ethanol/water 200 mL, CO_2_ or N_2_ 0.8 MPa, and 4 h.

Next, the influence of pressurized CO_2_ on the removal of components was investigated. Figure 3 compares the removal of components between treatments with CO_2_ and N_2_. It was clear that the addition of CO_2_ had little effect. The aim of adding CO_2_ was to promote the extraction and removal of components such as hemicellulose by providing acidic environment. The pH of water can be lowered to around three by dissolving CO_2_ (Mohammadian et al., 2023). Under the experimental conditions of this study, if all the acetyl groups in the rice husk were released as acetic acid, the concentration would be 33 mmol/L, corresponding to a pH of 3.1. Thus, the pH achievable by acidification using CO_2_ can be achieved solely by the acetyl groups. Pasquini et al. (2005) similarly attempted pre-treatment of sugar cane bagasse with ethanol/water under pressurized CO_2_ atmosphere, but the purpose was delignification by the use of CO_2_ as solvent under supercritical conditions. Little influence of CO_2_ in the present study is attributed to the acidification of the solvent even in the absence of CO_2_ by organic acids like acetic acid generated from hemicellulose, which does not allow for CO_2_ to contribute to further acidification. Moreover, solubility of CO_2_ in ethanol/water decreases with temperature (Dalmolin et al., 2006), limiting its contribution to the liquid phase reactions under employed conditions. Nevertheless, taking the global demand for developing CO_2_ utilization technologies into consideration, the absence of any negative effects of CO_2_, compared to N_2_, is valuable information. Since the acidification of water by CO_2_ dissolution is more pronounced at lower temperatures (Toor et al., 2011), the effect of pressurized CO_2_ may be obtained if the treatment is conducted at sufficiently low temperatures while suppressing the generation of organic acids.

### 3.2 Fractionation of four components in rice husk

**FIGURE 4:**
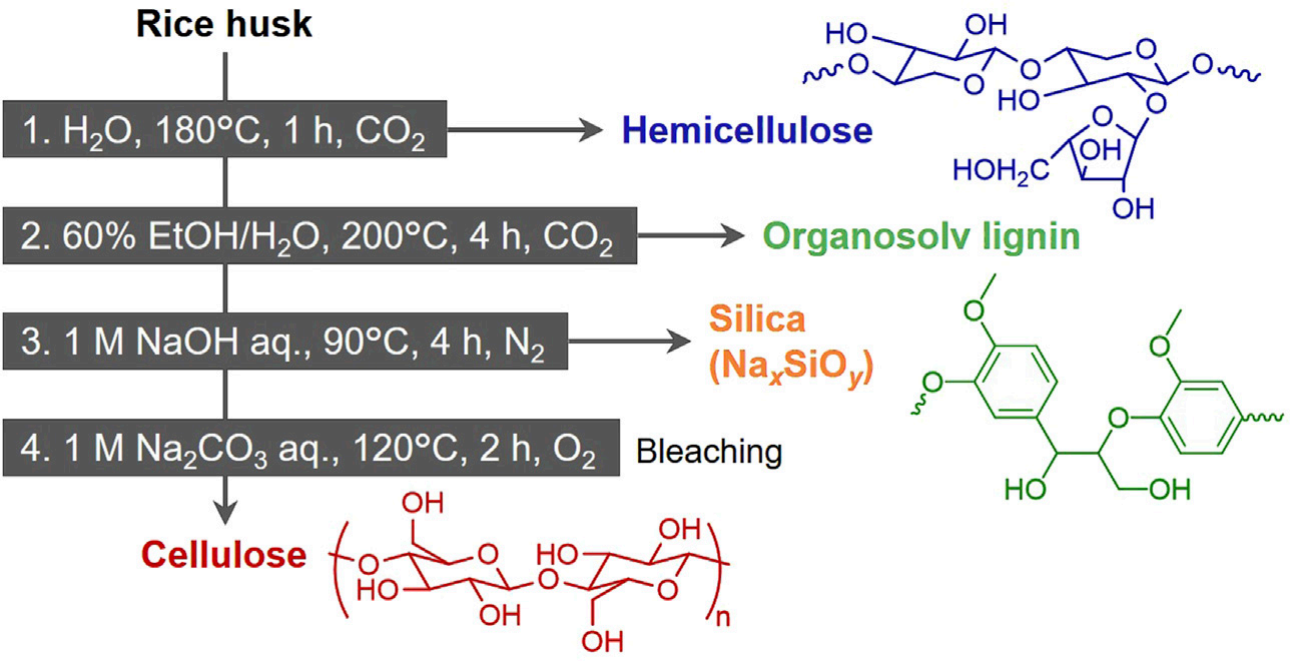
Proposed multi-step pre-treatment for recovering hemicellulose, lignin, silica and cellulose from rice husk.

**FIGURE 5:**
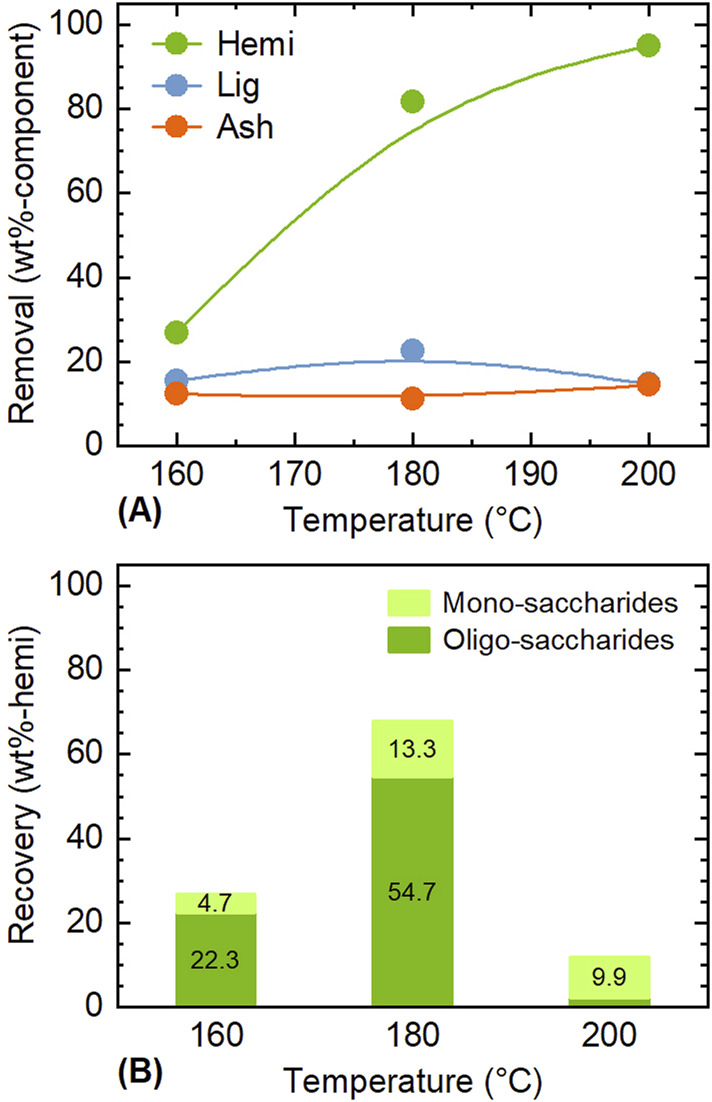
Influence of temperature on **(A)** removal of hemicellulose and **(B)** recovery of mono- and oligo-saccharides during the pre-treatment with water. Conditions: rice husk 10 g, water 200 mL, CO_2_ 0.8 MPa, and 1 h.

Based on the experimental results above, a method to separate the four components, including silica, is proposed in Figure 4. The first step is to separate hemicellulose with water as solvent, which proved effective in isolating nearly all hemicellulose while having minimal impact on other components. For completing the treatment with short time and achieving high extraction, higher temperature is more suitable; however, saccharides derived from hemicellulose are thermally unstable and may convert into furans, organic acids, and humins when excess treatment conditions are applied (Mohammadian et al., 2023; Ramos, 2003). To confirm this, the recovery of saccharides extracted during water treatment was analyzed (Figure 5). At 160°C, the recovery of saccharides in solvent was almost same with the removal of hemicellulose, but as the temperature increased, a difference between them emerged. At 200°C, the removal of hemicellulose was 95 wt%, while the recovery of saccharides (mono-saccharides + oligo-saccharides) was only 12.0 wt%. Therefore, 180°C was chosen as the treatment temperature suitable for recovering saccharides in the solvent. Notably, xylooligosaccharides derived from lignocellulosic biomass have attracted great attention for the potential to be used in food, feed, health, and cosmetics applications (Santibanez et al., 2021; Amorim et al., 2019). The degree of polymerization (DP) of oligo-saccharides obtained from hydrothermal extraction is generally required to be reduced to lower level such as DP2–DP4 by further hydrolysis, depending on the applications. For instance, low-DP xylooligosaccharides have higher prebiotic potential (Sukri and Mimi Sakinah, 2018).

The first treatment also removed water-soluble ash, primarily composed of AAEMs, contributing to the purification of silica in the ash. The subsequent treatment uses 60 vol% ethanol/water to remove lignin. The delignification at this step is incomplete as indicated in Figure 2. However, harsh extraction conditions give damage to or remove ash and cellulose. Therefore, the fourth treatment was added for bleaching and obtaining high-purity pulp instead.

Ideally, the main components remaining in the solid residue after the second treatment should be cellulose and ash (silica). To extract both as chemical raw materials, incineration should not be employed for obtaining silica. Therefore, it was determined to extract silica as silicate (Na_2*x*_Si_*y*_O_2*y+x*_) using an alkaline aqueous solution. The silica in rice husk is amorphous and can be extracted through this reaction (2*x*NaOH + *y*SiO_2_ → Na_2*x*_Si_*y*_O_2*y+x*_ + *x*H_2_O) under mild conditions. In preliminarily test, when silica extraction was attempted using 0.3 M NaOH aqueous solution under CO₂, the extraction rate was only 26.6 wt%. Since silica extraction with alkaline aqueous solutions is strongly influenced by the solvent’s pH (Hsieh et al., 2009; Park et al., 2021), this result was attributed to the pH reduction caused by dissolved CO₂ (2NaOH + CO_2_ → Na_2_CO_3_ + H_2_O). Indeed, the pH of the solution after the reaction was 8.3. Thus, N₂ was employed as the filling gas for this treatment. The obtained sodium silicate can be used as a raw material for a wide variety of silica-based materials, as well as in applications such as binders and emulsifiers (Minju et al., 2021; Liu and Ott, 2020).

The final treatment applies alkaline hydrothermal processing under an oxygen atmosphere to remove residual lignin. As mentioned earlier, oxygen is effective for lignin depolymerization and extraction, while its lower oxidation ability, compared to other oxidative bleaching method, such as the one using hydrogen peroxide (More et al., 2021; Bazargan et al., 2020), allows minimal loss of cellulose when applying appropriate conditions.

**FIGURE 6:**
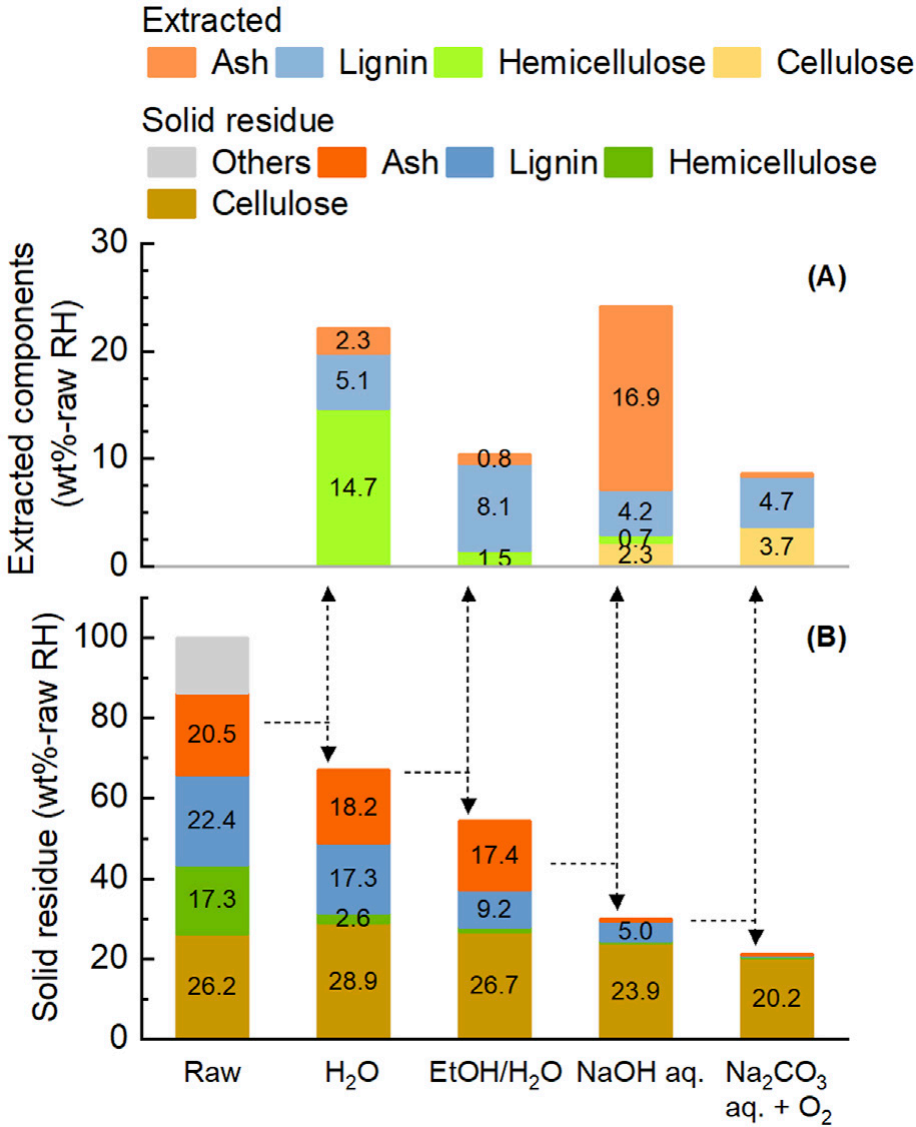
Yield of extracted components **(A)** and composition of solid residue **(B)** during the multi-step pre-treatment of rice husk. Initial rice husk 10 g, solvent 200 mL and filling gas 0.8 MPa. Other conditions are described in Figure 4.

The composition of solid residues and the yields of extracted components after sequential application of these four treatments to rice husk are shown in Figure 6. The total extraction rates after all treatments were 97.6 wt% for hemicellulose, 98.4 wt% for lignin, and 99.3 wt% for ash. Each stage selectively extracted the target component. Hemicellulose accounted for 66.5 wt% of the extracted components from the first treatment, lignin accounted for 78.1 wt% of the second extracts, and ash accounted for 77.5 wt% of the third extracts. Such a selective fractionation of the four components was attributable to the stepwise treatment under appropriate conditions. The loss of cellulose during the process leading to pulp was 22.8 wt%. The fourth treatment accounted for more than half portion of extracted cellulose.

**FIGURE 7:**
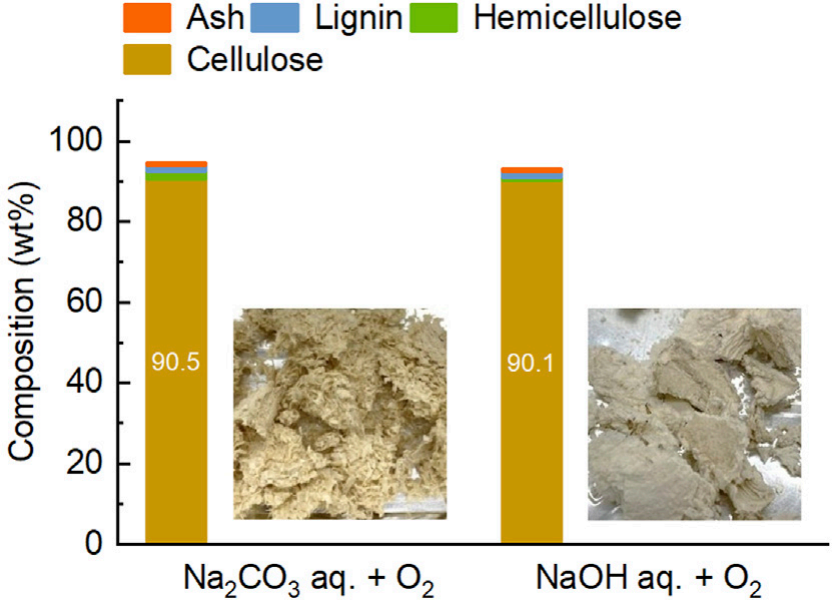
Comparison of 1 M Na_2_CO_3_ and 1 M NaOH aqueous solutions as solvents for the fourth step treatment. Images show pulps obtained from the treatment.

The final pulp obtained was of high purity, containing only 1.6 wt% lignin and 0.6 wt% ash (Figure 7). For comparison example, Dagnino et al. (2017) used a two-step treatment with dilute sulfuric acid and NaOH/water/ethanol to produce pulp from rice husk, which retained 6 wt% lignin and 24 wt% ash. In the present study, Na_2_CO_3_ aqueous solution was used as the solvent for the fourth treatment. Considering pH of the solution, NaOH potentially results in greater lignin removal. The performance of these salts is compared in Figure 7 using their solution at the same concentration. When used NaOH, the lignin content in the pulp decreased only slightly to 1.4 wt%, but the loss of cellulose increased to 26.6 wt%. Thus, lignin removal in the fourth treatment was mainly dependent on the presence of oxygen, and the advantage of using NaOH aqueous solution was considered minimal. Furthermore, NaOH aqueous solution is carbonated during processing of biomass (Kudo et al., 2014). Taking the regeneration and reuse of alkaline aqueous solution into account, Na_2_CO_3_ would be a reasonable choice for this treatment. As mentioned earlier, lignin in this treatment is oxidatively depolymerized and extracted. Consequently, organic acids such as oxalic acid, acetic acid, and formic acid are selectively obtained. However, many other organic compounds are also included in the extracts. Considering the low lignin content in the raw material of this step and the low concentration of lignin-derived compounds in the extract, the purpose of this treatment was set solely as cellulose purification. For this reason, compositional analysis of the extract was not performed in the present study. A method for valorizing the extracted organics is also discussed later.

In previous studies on biomass component fractionation, the main objective was to separate organic components, but there have been some reports that also considered the separation of ash. Zhang et al. (2015) applied a concept similar to that in the present study to separate rice husk into xylan, lignin, glucose-derived ethanol, and ash through hydrolysis, solvent extraction (butanediol), dilute acid hydrolysis, and then alcoholic fermentation, yielding 10, 22, 5, and 17 wt% from rice husk, respectively. Furthermore, Durand et al. (2024) recently proposed a sequential pre-treatment process for extracting biopolymers (cellulose and lignin) along with silica from rice husks, achieving lignin recovery at 47% with 82% purity and extraction of cellulose with 1% ash content and 82% purity. Compared to these prior studies, the originality of the present research includes pursuing the pre-treatment without expensive reagents including catalytic species and successfully separating each component while maintaining their structure as natural as possible. The introduction of oxidative alkaline treatment resulted in the production of very high-purity pulp. Hemicellulose-derived saccharides and cellulose were indeed obtained while moderately retaining their degree of polymerization (DP), ensuring their usability in broad applications. Further studies on this type of process and fine-tuning of treatment conditions are expected to enable even higher selectivity.

However, the proposed process has challenges to be solved: 1) Further optimization of processing conditions is essential to improve the yield of each extract. Many operational factors influence the yields. Since the performance of each treatment affects others, an overall process consideration is necessary. 2) Performing multi-step pre-treatment using solvents raises concerns about the significant energy required for heating the solution and materials. To address this, utilization of heat exchange is important. The residual heat from previous treatments can be used for the rice husk samples. While, heating of the solutions is recommended to proceed with heat exchange starting from the higher-temperature treatments. However, efficient heat exchange may be challenging with batch processing, as used in this study. A potential alternative is flow-through processing, where the extraction liquid flows through a fixed bed of rice husk samples. This system allows for easy heat exchange between the inflow and outflow solutions and enables all steps to be performed with a single reactor by changing the inflow solution and processing conditions. The flow-through extraction has recently gained attention for its ability to extract components like lignin in a natural structure-preserved form (Brandner et al., 2021; Xu et al., 2021). 3) From a cost perspective, solvent recycling is essential. For water, ethanol/water, and the aqueous solution used in the third step, their evaporation is necessary to recover the extracts and reuse the solution. If impurities like organic acids are present in the recovered solution and difficult to separate, it will be necessary to investigate their accumulation and impact on the treatments. An alternative to energy-intensive evaporation-based separation is the application of membrane separation technology. In the fourth step, the extract solution contains a mixture of organic compounds such as organic acids. Their recovery along with regeneration of the solution should be considered when they are valuable. Alkaline hydrothermal gasification is considered to be a method for regenerating the alkaline solution. It has been reported that the reaction using solid catalysts enabled to recover high-calorie gas consisted mainly of methane from lignin and Na_2_CO_3_ aqueous solution free from organic matter (Kudo et al., 2014). 4) For the practical implementation of the process, it is necessary to ensure that the internal pressure is sufficiently low. For example, in Japan, if the operating pressure exceeds 1 MPa, the system is considered to contain high-pressure gas, raising regulatory hurdles for process implementation. The saturated vapor pressure of water increases rapidly within the temperature range targeted in this study, exceeding 1 MPa at temperatures above 180°C. Therefore, it is essential to operate at the lowest feasible temperature and keep the pressure of any filling gas as low as possible.

## 4 Conclusion

This study demonstrates a multi-step pre-treatment process for the fractionation of rice husk into its primary components: hemicellulose, lignin, cellulose, and silica. The performance of ethanol/water pre-treatment was initially assessed due to the attractiveness in the use of only green solvents. Based on the results, the four-step pre-treatment was proposed, incorporating alkaline aqueous treatments. By systematically integrating those treatments, the process achieved their selective separation, overcoming limitations of one-step methods. The hydrothermal step effectively extracted hemicellulose (66.5 wt% in the extract), preserving its functional integrity as saccharides for subsequent applications. Organosolv treatment optimized lignin removal (78.1 wt% in the extract) using ethanol/water mixtures, a solvent chosen for its environmental compatibility and efficacy. The treatment with NaOH aqueous solution under nitrogen enabled silica extraction (77.5 wt% in the extract) as sodium silicate, a versatile industrial precursor. The final alkaline hydrothermal treatment with O_2_ achieved effective lignin removal, producing high-purity cellulose that included only 1.6 wt% lignin and 0.6 wt% ash, suitable for diverse applications. CO_2_ was applicable as a filling gas for the first two treatments, although no positive effect was observed, compared to inert N_2_. This strategy highlights the potential of rice husk as a versatile feedstock for both organic and inorganic materials, paving the way for broader industrial applications. Future work should focus on further tuning pre-treatment conditions and evaluating economic viability to facilitate practical implementation.

## Data Availability

The original contributions presented in the study are included in the article, further inquiries can be directed to the corresponding author.
